# Prevalence and risk factors of toxigenic *Clostridioides difficile* asymptomatic carriage in 11 French hospitals

**DOI:** 10.3389/fmed.2023.1221363

**Published:** 2023-07-19

**Authors:** Sarah Jolivet, Jeanne Couturier, Patrick Grohs, Aurélie Vilfaillot, Jean-Ralph Zahar, Pierre Frange, Anne Casetta, Véronique Moulin, Christine Lawrence, Patricia Baune, Cléo Bourgeois, Axel Bouffier, Claudine Laussucq, Lydia Sienzonit, Simon Picard, Isabelle Podglajen, Najiby Kassis-Chikhani, Frédéric Barbut

**Affiliations:** ^1^Unité de prévention du risque infectieux, Hôpital Saint Antoine, Paris, France; ^2^Laboratoire de microbiologie de l’environnement, Hôpital Saint Antoine, Paris, France; ^3^National Reference Laboratory for Clostridioides difficile, Paris, France; ^4^Laboratoire de microbiologie, Hôpital Européen Georges Pompidou, Paris, France; ^5^Unité de Recherche Clinique, Hôpital Européen Georges Pompidou, Paris, France; ^6^INSERM Centre d’Investigation Clinique 1418, Paris, France; ^7^Unité de Prévention du Risque infectieux, Hôpitaux Avicenne, Bobigny/Jean Verdier, Bondy/René Muret, Sevran, France; ^8^Équipe de Prévention du Risque infectieux, Laboratoire de microbiologie clinique, Hôpital Necker – Enfants malades, Groupe hospitalier Assistance Publique – Hôpitaux de Paris (APHP) Centre – Université Paris Cité, Paris, France; ^9^Équipe de Prévention du Risque infectieux, Hôpital Cochin, Paris, France; ^10^Équipe de Prévention du Risque infectieux, Hôpitaux Corentin Celton/Vaugirard, Issy-les-Moulineaux, France; ^11^Équipe de Prévention du Risque infectieux, GHU Paris-Saclay site R. Poincaré, Garches, France; ^12^Équipe de Prévention du Risque infectieux, Hôpital Paul Brousse, Villejuif, France; ^13^Équipe de Prévention du Risque infectieux, Hôpital Européen Georges Pompidou, Paris, France

**Keywords:** *Clostridioides difficile*, colonization, asymptomatic, prevalence, risk factors

## Abstract

*Clostridioides difficile* infection (CDI) incidence has increased over the last 20 years. Studies suggest that asymptomatic carriers may be an important reservoir of *C. difficile* in healthcare settings. We conducted a point prevalence study to estimate the toxigenic *C. difficile* asymptomatic carriage rate and the associated risk factors in patients >3 years old. Between September 16, 2019 and January 15, 2020, all patients hospitalized in 11 healthcare facilities in the Paris urban area were included in the study. They were screened on the day of the survey for toxigenic *C. difficile* carriage by rectal swab and interviewed. Isolates were characterized by PCR ribotyping and multiplex PCR targeting toxin genes. A logistic regression model was used to determine the risk factors associated with toxigenic *C. difficile* asymptomatic carriage using uni- and multivariate analysis in the subpopulation of patients >3 years old. During the study period, 2,389 patients were included and screened. The median age was 62 years (interquartile range 35–78 years) and 1,153 were male (48.3%). Nineteen patients had a previous CDI (0.9%). Overall, 185/2389 patients were positive for *C. difficile* (7.7%), including 93 toxigenic strains (3.9%): 77 (82.8%) were asymptomatic (prevalence 3.2%) whereas 12 (12.9%) were diarrheic. Prevalences of toxigenic *C. difficile* were 3.5% in patients >3 years old and 7.0% in ≤3 years old subjects, respectively. Toxigenic strains mainly belonged to PCR ribotypes 106 (*n* = 14, 15.0%), 014 (*n* = 12, 12.9%), and 020 (*n* = 10, 10.8%). Among toxigenic strains, 6 (6.4%) produced the binary toxin. In multivariate analysis, two factors were positively associated with toxigenic *C. difficile* asymptomatic carriage in patients >3 years old: multidrug-resistant organisms co-carriage [adjusted Odd Ratio (aOR) 2.3, CI 95% 1.2–4.7, *p* = 0.02] and previous CDI (aOR 5.8, CI 95% 1.2–28.6, *p* = 0.03). Conversely, consumption of raw milk products were associated with reduced risk of toxigenic *C. difficile* colonization (aOR 0.5, CI 95% 0.2–0.9, *p* = 0.01). We showed that there was a low prevalence of asymptomatic toxigenic *C. difficile* carriage in hospitalized patients. Consumption of raw milk prevents toxigenic *C. difficile* colonization, probably due to the barrier effect of milk-associated bacteria.

## 1. Introduction

*Clostridioides difficile* is the leading cause of healthcare-associated diarrhea, accounting for almost half of all nosocomial gastrointestinal infections in hospitals in Europe (1). Exposure to *C. difficile* can lead to asymptomatic carriage or *C. difficile* infection (CDI) with a wide range of clinical presentations and outcomes (from mild diarrhea to severe colitis and death). CDI is associated with a substantial morbidity and mortality. In France, the extra cost of CDI in public acute-care hospitals was estimated to 163.1 million per year (2). Prevention of CDI stills remains challenging in both acute care and long-term care facilities. The CDI incidence in hospitalized patients has increased over the last 20 years in France (3). The asymptomatic *C. difficile* colonization rate is frequent although highly variable across studies (range: 6–18%) (4–8). Main risk factors for asymptomatic *C. difficile* carriage included previous antibiotic treatment, use of gastric acid suppression therapy, prior hospitalization and history of CDI (4, 9, 10). Risk factors described in the literature vary widely, depending especially on the sampling technique, the *C. difficile* detection method, the population targeted and the epidemiology situation (outbreak versus endemicity). Colonization is a prerequisite to infection, and asymptomatic carriage is associated with a higher risk of CDI (11). Moreover, asymptomatic carriers of toxigenic strains are an important and hidden reservoir of *C. difficile* in healthcare settings and could play a key role in the transmission of this spore-forming micro-organism (12). A recent study showed that the environment of asymptomatic *C. difficile* carriers is as contaminated as that of symptomatic CDI patients (13). A quasi-experimental controlled study revealed that identification and isolation of *C. difficile* carriers was associated with a decreased incidence of CDI (14). However, screening asymptomatic carrier is not currently recommended in infection control guidelines (15). The aim of the study was to evaluate the prevalence of toxigenic *C. difficile* asymptomatic carriage and its associated risk factors in order to provide insights into this potential reservoir in healthcare facilities.

## 2. Materials and methods

### 2.1. Study design and setting

The study was performed in 11 sites of Assistance Publique-Hôpitaux de Paris (AP-HP) providing intensive care, acute care for adults, maternity, pediatrics, geriatric and long-term care with a total capacity of 6,454 beds: Hôpital Européen Georges Pompidou (EGP01; acute care), Avicenne (AV02; acute care), Jean Verdier (JV03; maternity and acute care), René Muret (RM04; geriatric long stay), Corentin Celton (CC05; geriatric long stay and reeducation), Vaugirard-Gabriel Pallez (VG06; geriatric long stay), Necker-Enfants malades (NCK07; maternity and acute care for adults and children), Raymond Poincaré (RP08; acute care), Saint-Antoine (SA09; acute care), Cochin (CCH10; maternity and acute care) and Paul Brousse (PB11; liver transplant and geriatrics). This study is an ancillary study of the project CODBAHRE (16), a point prevalence study to estimate the multidrug-resistant organisms (MDRO) fecal carriage rate.

This study was approved by agreement with French regulations (AP-HP project CODBAHRE no. 180561; IDRCB no. 2019-A01226-51).

We conducted a serial cross-sectional survey of *C. difficile* carriage between September 16, 2019 and January 15, 2020. All patients hospitalized more than 24 h on a given day in one of the 11 facilities (except patients from psychiatric units) who agreed to participate in the survey were eligible to inclusion. A non-opposition form was obtained from each patient included in the study (or from parents/guardians for pediatric patients). Clinical wards and eligible patients were informed by information notes according to the protocol.

Six nurses were specifically recruited and trained to implement the protocol. They interviewed the patients and collected the data. The questionnaire (available as Supplementary Table S1) was completed by the nurses and included demographic data, life conditions, previous treatments and food consumption.

For all included patients, a screening sample was taken using an eSwab^®^ system (COPAN, Brescia, Italy) by the nurse or by the patients themselves. The sample was collected by rectal swabbing, by soaking the swab in the stools or by swabbing an ostomy. All samples were sent to a central microbiology laboratory for subsequent analysis.

### 2.2. *Clostridioides difficile* isolation, strain characterization and typing

All samples were plated on the selective chromogenic medium ChromID^®^ *C. difficile* agar (bioMérieux^®^, Marcy-l’Etoile, France). Plates were incubated for 48 h at 37°C in an anaerobic atmosphere. Suspicious colonies (based on black coloration and the morphological aspect) were analyzed using matrix assisted laser desorption ionization time-of-flight (MALDI-TOF) mass spectrometry (Brucker Daltonics, Germany). DNA extraction was performed from colonies grown on Brucella agar (bioMérieux^®^, Marcy-l’Etoile, France) using the InstaGene Matrix^™^ kit (Bio-Rad^®^, Marnes-la-Coquette, France). Strains were characterized by a previously described multiplex PCR assay detecting *tpi*, *tcdA*, *tcdB*, *tcdC*, *cdtA*, and *cdtB* genes coding for the triose phosphate isomerase, toxin A, toxin B, TcdC and the two components of the binary toxin, respectively (17).

Isolates were typed by capillary gel electrophoresis-based PCR ribotyping on an ABI 3500 sequencer (Applied Biosystems, Foster City, CA, United States), using primers described by Bidet et al. (18). After DNA amplification, each PCR product was diluted at 1/200. One microliter of this dilution was mixed with 10.5 μL formamide and 0.5 μL GeneScan LIZ600 (Applied Biosystems^®^, Foster City, United States). The banding patterns were analyzed with the GeneMapper software (Thermo Fisher Scientific, Villebon-sur-Yvette, France). PCR ribotypes (RT) were assigned using the Webribo database.^1^

ESwab^®^ samples were also plated onto specific chromogenic culture media: chromID^®^ CARBA SMART, chromID^®^ VRE, and chromID^®^ ESBL-PE (bioMérieux, Craponne, France) to search for the presence of MDRO co-carriage.

### 2.3. Statistical analysis

Prevalence of *C. difficile* carriage (including toxigenic and non-toxigenic strains) was reported in the overall study population, by hospitals, by specialties, by age and by time from admission. Results were expressed as median [interquartile range (IQR)] for continuous variables and N (%) for categorical variables.

Risk factors for toxigenic *C. difficile* asymptomatic carriage in patients >3 years old were investigated by uni- and multivariate analysis. Asymptomatic patients with toxigenic *C. difficile* were compared with asymptomatic non- *C. difficile* carriers in patient >3 years old. Asymptomatic patients with toxigenic *C. difficile* was defined as patient without diarrhea (<3 stools per day for 48 h) and with a rectal sample positive for a toxigenic *C. difficile* isolate. Patients with diarrhea on the day of inclusion were excluded from the analysis. A logistic regression analysis was used to determine the risk factors associated with asymptomatic carriage of toxigenic *C. difficile*. Variables with a *p* < 0.20 in univariable analysis were included in the multivariate logistic regression model. A backward stepwise approach was used to identify independent predictors. Adjusted odds ratios (aOR) and 95% confidence intervals (95% CIs) were calculated for all variables. *p* < 0.05 were considered statistically significant. Statistical analysis was made using Stata^®^ software, v15 (StataCorp, College Station, Texas, United States).

## 3. Results

### 3.1. Description of the population

**Figure 1:**
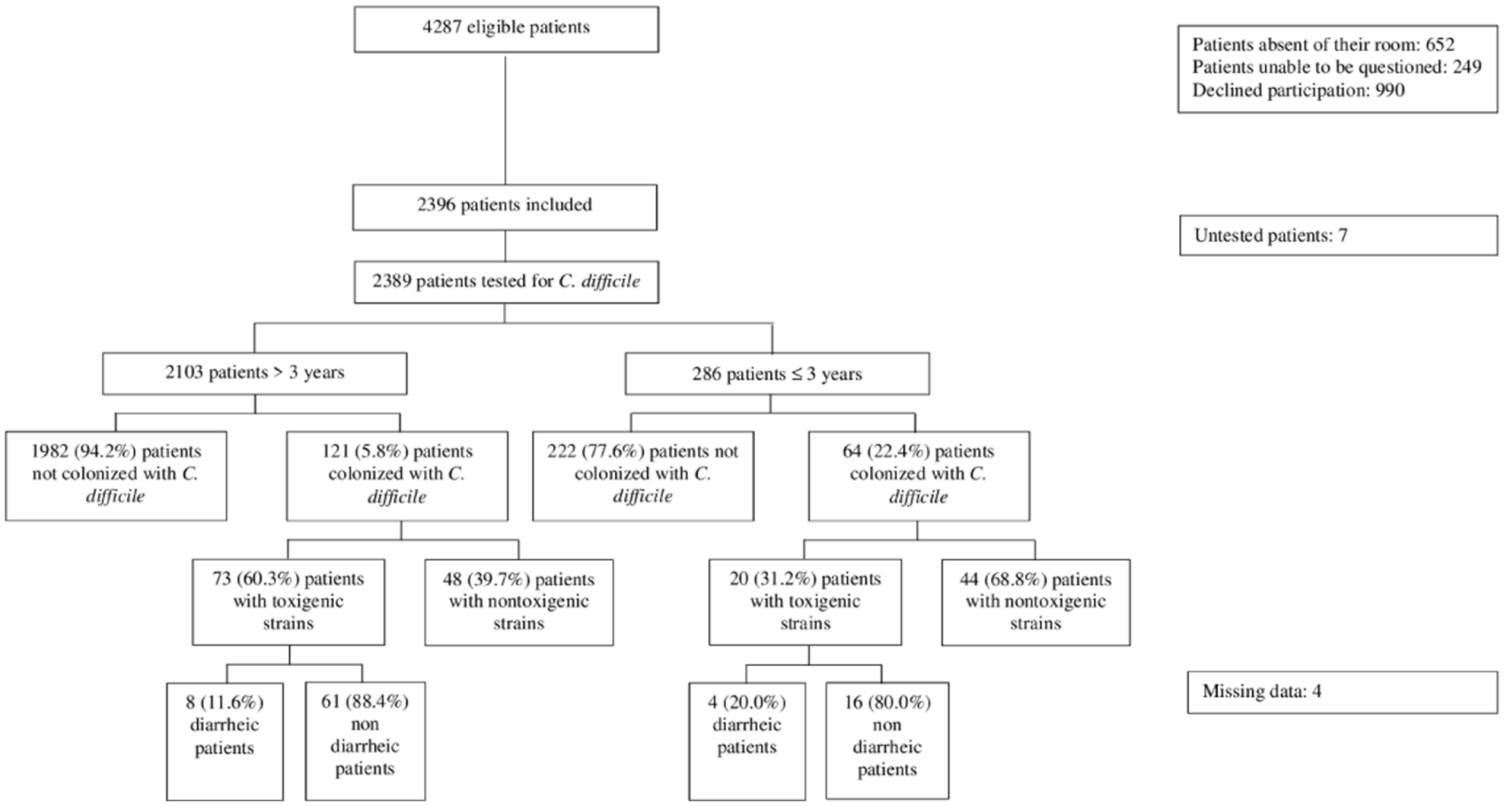
Flow chart of patient recruitment.

In the 11 participating hospitals on the day of the study, 4,287 patients were eligible, 2,396 patients were included (55.9%) and 2,389 were tested for *C. difficile* carriage (Figure 1). In total, 1,193 (50.4%) swabs were collected by patients themselves and 1,176 (49.6%) by nurses. The median age was 62 years (IQR 35–78 years, range 0–106 years), 1,153 patients (48.3%) were male and 2,317 (97.1%) were born in France. Comorbidities were frequent (*n* = 1,599, 68.2%); in particular, 922 (38.5%) patients had a cardiopathy, 384 (16.1%) were diabetic, 309 (12.9%) had a progressive cancer, 206 (8.6%) had a chronic respiratory failure, 199 (8.3%) had a chronic kidney failure, 137 (5.7%) had a progressive hemopathy, 75 (3.1%) were transplants, 59 (2.5%) had a cirrhosis, 22 (0.9%) had HIV and 18 (0.7%) were under dialysis. One thousand seventy-one (45.7%) patients had a history of hospitalization during the last 12 months, mostly in France (*n* = 1,036, 44.2%). Four hundred fifty-four (19.6%) traveled abroad in the previous 3 months. Overall, 1,074 (48.4%) patients had received antibiotics during the last 6 months. Nineteen (0.9%) patients reported a previous episode of CDI.

### 3.2. Characterization of *Clostridioides difficile* strains

Overall, 185 (7.7%) samples were positive for *C. difficile* including 93 (50.3%) toxigenic strains and 92 (49.7%) nontoxigenic strains. The most prevalent toxigenic RT were 106 (*n* = 14, 15.0%), 014 (*n* = 12, 12.9%), and 020 (*n* = 10, 10.8%). Six of the 14 RT 106 strains were isolated in samples from the NCK07 healthcare facility, from patients <1 year old (*n* = 4), 3 years old (*n* = 1) and 14 years old (*n* = 1), hospitalized in different wards. Among toxigenic strains, 6 (6.4%) were tested positive for the binary toxin genes *ctdA* and *cdtB* and belonged to RT 126 (*n* = 3), 078 (*n* = 2), and 023 (*n* = 1). The most frequent nontoxigenic strains were 039 (*n* = 28, 30.4%), 205 (*n* = 20, 21.7%), and 010 (*n* = 17, 18.5%). The distribution of RT is described in the Table 1.

**Table 1:** Distribution of the PCR-ribotypes of the 185 *Clostridioides difficile* strains.

	PCR-ribotype	N strains
Toxigenic strains (*n* = 93)	001	2
	002	5
	003	2
	012	4
	014	12
	015	5
	017	1
	018	1
	020	10
	023	1
	029	2
	046	1
	078	2
	081	1
	087	1
	106	14
	126	3
	211	2
	220	2
	241	1
	446	2
	559	2
	590	1
	651	3
	739	3
	AI-18	1
	AI-21/0	1
	AI-84	3
	AI-9-1	1
	Other	4
Nontoxigenic strains (*n* = 92)	009	8
	010	17
	019	1
	031	1
	039	28
	067	1
	085	1
	205	20
	557	2
	593	2
	715	3
	Other	8

### 3.3. Prevalence of toxigenic *Clostridioides difficile* carriage

Of 93 patients harboring a toxigenic *C. difficile* strain, 77 (82.8%) were asymptomatic whereas 12 (12.9%) were diarrheic (Figure 1). Among the 12 diarrheic patients, 8 were > 3 years old. The strains isolated from those 8 patients belonged to RT 078, 046, AI-21/0, 106, 015, 211, 014 and 126. Moreover, among toxigenic *C. difficile* carriers, 18 patients were screened within 3 days of admission.

Prevalences of *C. difficile* and toxigenic *C. difficile* were 5.8% (121/2103) and 3.5% (73/2103) in patients >3 years old, respectively; and 22.4% (64/286) and 7.0% (20/286) in youngest subjects, respectively. *C. difficile* carriage ranged from 2.6% (2/76) to 20.2% (68/336) depending on the hospital, and that of toxigenic strain from 1.0% (3/307) to 7.4% (25/336) (Table 2). The prevalence of *C. difficile* carriage varied also according to the type of ward, from 0.7% (1/141) in motherhood to 23.4% (40/171) in pediatrics, with rates of 0.7% (1/141) and 9.9% (17/171) respectively for toxigenic *C. difficile* carriage. The prevalence of toxigenic *C. difficile* in patient hospitalized ≤3 days and > 3 days were 2.3% (18/769) and 4.6% (73/1578) respectively.

**Table 2:** Frequencies of *Clostridioides difficile* and toxigenic *C. difficile* carriers by hospital and by ward.

	Patients	*C. difficile* carriage		Toxigenic *C. difficile* carriage	
	n	n	%	n	%
Hospital					
EGP01	433	23	5.3	17	3.9
AV02	265	17	6.4	10	3.8
JV03	92	6	6.5	3	3.3
RM04	76	2	2.6	2	2.6
CC05	180	10	5.6	6	3.3
VG06	90	5	5.6	4	4.4
NCK07	336	68	20.2	25	7.4
RP08	134	13	9.7	7	5.2
SA09	402	24	6.0	15	3.7
CCH10	307	12	3.9	3	1.0
PB11	74	5	6.8	1	1.3
Ward					
Medical	1,085	66	6.1	35	3.2
Surgery	263	15	5.7	10	3.8
Motherhood	141	1	0.7	1	0.7
Nursling	203	35	17.2	9	4.4
Pediatrics	171	40	23.4	17	9.9
Adult intensive care unit	129	8	6.2	6	4.6
Long-term care facilities	390	18	4.6	13	3.3
Unknow	7	2	28.6	2	28.6
Age					
[0–3] years	286	64	22.4	20	7.0
]3–18] years	94	12	12.8	6	6.4
]18–45] years	409	17	4.2	11	2.7
]45–65] years	528	32	6.1	19	3.6
>65 years	1,072	60	5.6	37	3.4
Time from admission					
[0–3] days	769	37	4.8	18	2.3
]3–7] days	522	42	8.1	22	4.2
>7 days	1,056	104	9.8	51	4.8
Unknow	42	2	4.8	2	4.8
**Total**	**2,389**	**185**	**7.7**	**93**	**3.9**

### 3.4. Risk factors for asymptomatic toxigenic *Clostridioides difficile* carriage in patients >3 years old

Risk factors for toxigenic *C. difficile* asymptomatic carriage in patients >3 years old were investigated by uni- and multivariate analysis: 61 toxigenic *C. difficile* asymptomatic carriage patients were compared with 1724 asymptomatic non-carriers of *C. difficile* (Table 3).

**Table 3:** Univariate and multi-variate logistic regression analyses of factors associated with toxigenic *Clostridioides difficile* asymptomatic carriers aged >3 years.

Variables	Asymptomatic non-*C. difficile* carriers *n* =1,724	Asymptomatic toxigenic *C. difficile* carriers *n* = 61	OR (95% CI) Univariate analysis	*P*-value	aOR (95% CI) Multivariate analysis	*P*-value
Age, years (median, IQR)	66 (47–80)	66 (45–78)	1.0 (1.0–1.0)	0.44		
Male gender	813 (47.2)	37 (60.7)	1.7 (1.0–2.9)	**0.04**		
Hospital						
EGP01	347 (20.1)	15 (24.6)		0.62		
AV02	214 (12.4)	9 (14.8)	1.0 (0.4–2.3)			
JV03	47 (2.7)	2 (3.3)	1.0 (0.2–4.4)			
RM04	71 (4.1)	2 (3.3)	0.7 (0.1–2.9)			
CC05	149 (8.6)	6 (9.8)	0.9 (0.3–2.4)			
VG06	76 (4.4)	4 (6.6)	1.2 (0.4–3.8)			
NCK07	115 (6.7)	7 (11.5)	1.4 (0.6–3.5)			
RP08	103 (6.0)	4 (6.6)	0.9 (0.3–2.8)			
SA09	313 (18.2)	9 (14.8)	0.7 (0.3–1.5)			
CCH10	226 (13.1)	2 (3.3)	0.2 (0.0–0.9)			
PB11	63 (3.7)	1 (1.6)	0.4 (0.0–2.8)			
Hospital type						
Acute care	1,313 (76.2)	42 (68.9)		0.28		
Geriatric/Long-term care	296 (17.2)	12 (19.7)	1.3 (0.7–2.4)			
Pediatric	115 (6.7)	7 (11.5)	1.9 (0.8–4.3)			
Ward type						
Medical	862 (50.0)	30 (49.2)		0.42		
Surgery	211 (12.2)	8 (13.1)	1.1 (0.5–2.4)			
Motherhood	134 (7.8)	1 (1.6)	0.2 (0.0–1.6)			
Pediatrics	69 (4.0)	5 (8.2)	2.1 (0.8–5.5)			
Adult Intensive care unit	112 (6.5)	5 (8.2)	1.3 (0.5–3.4)			
Long-term care facilities	336 (19.5)	12 (19.7)	1.0 (0.5–2.0)			
Origin of the patient						
Home	1,343 (78.1)	48 (78.7)		0.91		
Transfer	377 (21.9)	13 (21.3)	1.0 (0.5–1.8)			
Other patient characteristics						
France residing	1,682 (97.6)	59 (96.7)	0.7 (0.2–3.1)	0.68		
Hospitalization during the last 12 months	759 (44.6)	33 (54.1)	1.5 (0.9–2.4)	0.15		
Traveling abroad	369 (21.6)	8 (13.1)	0.5 (0.3–1.2)	0.11		
Antimicrobial treatment during the last 6 months	763 (47.8)	29 (59.2)	1.6 (0.9–2.8)	0.12		
Comorbidity	1,229 (72.7)	52 (88.1)	2.8 (1.3–6.2)	**0.01**		
Proton pump inhibitor	683 (39.7)	26 (42.6)	1.1 (0.7–1.9)	0.64		
Antacids	115 (6.7)	7 (11.5)	1.8 (0.8–4.1)	0.15		
Laxatives	587 (34.3)	24 (40.7)	1.3 (0.8–2.2)	0.31		
Previous CDI	10 (0.7)	2 (4.5)	6.8 (1.4–32.1)	**0.01**	5.8 (1.2–28.6)	**0.03**
MDRO co-carriage in this study	253 (14.7)	18 (29.5)	2.4 (1.4–4.3)	**0.002**	2.3 (1.2–4.7)	**0.02**
Home made products consumption	1,337 (78.5)	45 (73.8)	1.3 (0.7–2.3)	0.38		
Vegan or vegetarian	56 (3.3)	5 (8.3)	2.7 (1.0–6.9)	**0.04**		
Raw milk products consumption	1,012 (59.8)	26 (44.8)	0.5 (0.3–0.9)	**0.02**	0.5 (0.2–0.9)	**0.01**

aOR, adjusted Odd Ratio; CDI, *Clostridioides difficile* infection; CI, confidence intervals; IQR, interquartile range; MDRO, multidrug-resistant organisms; OR, Odd Ratio. Significant values are indicated in bold.

In univariate analysis, six factors were significantly associated with toxigenic *C. difficile* asymptomatic carriage: male gender, comorbidity, previous CDI, MDRO co-carriage, vegan or vegetarian and consumption of raw milk products. Other factors, e.g., previous antimicrobial treatment, use of proton-pump inhibitor, laxatives or anti acids or hospitalization during the last 12 months were more frequently found in *C. difficile* carriers than controls but the difference was not significant.

In multivariate analysis, only two factors remained significantly associated with toxigenic *C. difficile* asymptomatic carriage: MDRO co-carriage (aOR 2.3, CI95% 1.2–4.7, *p* = 0.02) and previous CDI (aOR 5.8, CI95% 1.2–28.6, *p* = 0.03). Conversely, consumption of raw milk products were associated with reduced risk of toxigenic *C. difficile* colonization (aOR 0.5, CI95% 0.2–0.9, *p* = 0.01).

## 4. Discussion

In this large French multicenter point-prevalence study, a low prevalence of toxigenic *C. difficile* carriage was observed among hospitalized patients (3.9%). These results provide valuable epidemiological insight into the understanding of CDI, a frequent and severe condition in hospitalized patients. Asymptomatic carriers of *C. difficile* have been shown to play a role in the transmission of CDI (11, 19) and can contaminate the hospital environment by spores. Moreover, asymptomatic carriers after discharge from hospital could also be a source of community-associated CDI cases (20, 21).

Our results are in the lower range of asymptomatic carriage previously reported in hospitalized patients (6–18%) and also lower than the prevalence of 5.8% found in another recent French multicenter study (4–8). This result may reflect differences in studied populations, or local epidemiology such as hospital outbreaks. It can also be explained by different microbiological techniques used to isolate *C. difficile*. We used rectal swabbing and direct culture whereas others used stool samples and enriched culture (4).

Children ≤3 years old were frequently found positive for *C. difficile* (22.4%) including with toxigenic *C. difficile* (7.0%) but the presence of *C. difficile* in this population has often been considered as non-pathogenic (22). Our results are in agreement with previous studies where a high colonization rate in children less than 2 years old was observed, ranging from 17 to 70% and mainly due to nontoxigenic strains (22–26). In contrast, prevalence was the lowest in parturient (0.7%), reflecting asymptomatic carriage in the community (27).

We identified two factors positively associated with toxigenic *C. difficile* carriage in subjects >3 years old. A previous CDI episode was already known as a risk factor (28, 29), and had the highest impact in our study (aOR 5.7, CI95% 1.2–28.1, *p* = 0.03). The other associated factor was MDRO co-carriage. This risk factor may be a marker of a prolonged hospitalization and antibiotic exposure. It may also reflect the high level of healthcare worker contacts linked to inter-individual transmission of pathogens.

In contrast, consumption of raw milk products reduced risk of toxigenic *C. difficile* carriage, probably due to the barrier effect of milk-associated bacteria. A meta-analysis suggested that probiotic prophylaxis may be a useful and safe CDI prevention strategy (30).

Another striking finding of this study was the lack of significant association between *C. difficile* colonization and antimicrobials or proton-pump inhibitor therapy. Use of antibiotics and proton-pump inhibitors, by disrupting the intestinal microbiota, were previously associated with *C. difficile* colonization and infection (31, 32). However, in a recent meta-analysis including 1,588 asymptomatic patients colonized with *C. difficile*, antibiotics in the previous 3 months was not associated with significant effects on risk of colonization (9).

The results of the strain typing analysis indicated a large diversity of strains and the distribution of RT was consistent with previous investigations. For several years, RTs 014 and 020 have been predominant in epidemiological studies in France or in Europe. They represented 18.7 and 21.9% of *C. difficile* strains in France in 2009 (33) and in 2024–2015 (34), respectively. They were also the most common RTs in the ECDC-coordinated surveillance of healthcare-associated CDI in 2016–2017 (35) and RT 014 was the third most frequent in a recent European study including 119 sites in 12 countries (36). In a recent French multicenter study, RT 014/020 was the most common toxigenic strain in *C. difficile* carriers (4). RT 106 is an emerging RT: between 2012 and 2017, its prevalence in France increased from <1 to 4.65% (3). The predominance of RT 106 in our study could be linked to a potential outbreak in one of the included healthcare facilities. However, epidemiological data to support this hypothesis are lacking. The absence of any RT 027 in our study should be noted. This hypervirulent strain was responsible for several outbreaks in France in the 2000s (37). Since then, control measures were implemented to prevent its spreading in Europe. In France, the frequency of RT 027 decreased from 21.7 to 9.56% between 2012 and 2017 (3). Our results support the successful control of the RT 027 spread in France.

This study has some limitations. First, the cross-sectional survey design did not allow us to investigate the dynamics of *C. difficile* colonization (acquisition, persistence and clearance of carriage). Second, generalizing our results is difficult because only 55.7% of eligible patients were included and screened. Third, the screening method for carriage was not the most sensitive since we used rectal swabbing rather than stool specimens and there was no broth enrichment step during culture.

A strength of our study was the inclusion of a large number of hospitalized patients from 11 facilities, without selecting patients at hospital admission or from high-risk units, thus reducing the impact of center effect. We also explored many risk factors including food consumption in the context of one health.

## 5. Conclusion

In conclusion, the prevalence of *C. difficile* toxigenic carriage was low in patients >3 years old (3.5%), but higher in ≤3 years old (7.0%) and in patients hospitalized >3 days (4.6%). These patients and may therefore represent a potential reservoir for CDIs.

## Data Availability

The raw data supporting the conclusions of this article will be made available by the authors, without undue reservation.

## Footnotes

^1^ https://webribo.ages.at/
