# Supplementary material for: Prevalence and risk factors of toxigenic Clostridioides difficile asymptomatic carriage in 11 French hospitals
Source: Front Med (Lausanne). 2023 Jul 19;10:1221363. doi: 10.3389/fmed.2023.1221363 (PMC10402895; doi:10.3389/fmed.2023.1221363)
Supplement: Supplementary file 1 [file Data_Sheet_1.docx]

Patient Questionnaire

**Protocol CODBAHRE**

**Patient consent 🞎 Date 🞎**

PATIENT IDENTIFICATION FOR RESEARCH

1. **Patient data:**

First name + Name:

Date of birth: Sex: F 🞎 M **🞎**

🕿:

Main country of residence:

If France, country of residence:

Country of secondary:

Hospitalization: Name of hospital: Admission date:

| Oncology |  | Hematology |  | Pediatrics * |  |
| --- | --- | --- | --- | --- | --- |
| Cardiology |  | Hepatology |  | General pediatrics |  |
| Heart surgery |  | Immunology |  | Pulmonology |  |
| Maxillofacial surgery |  | Infectious diseases |  | Surgical intensive cares |  |
| Orthopedic surgery |  | Internal Medicine |  | Medical intensive cares |  |
| Visceral surgery |  | Day hospital |  | Rheumatology |  |
| Dermatology |  | Nephrology |  | Non geriatric rehabilitation |  |
| Endocrinology |  | Neonatology |  | Geriatrics long term cares facilities |  |
| Geriatric house |  | Neurosurgery |  | Non geriatric long term cares facilities |  |
| Gastroenterology |  | Neurology |  | Emergency |  |
| Acute geriatrics |  | Ophthalmology |  |  |  |
| Gynecology - Obstetrics-Maternity |  | Otho-rhino-Laryngology |  |  |  |

**Other medical specialties:**

Origin of the patient before hospitalization:

Home 🞎 Transfer from another hospital: metropolitan France🞎 DOM TOM 🞎 abroad 🞎

If transfer, Country: Town:

Medical ward:

Medicine (adult or paediatric) 🞎 Surgery (adult or paediatric) 🞎 Obstetric 🞎 Non geriatric rehabilitation 🞎 Non geriatric long term cares facilities 🞎 Geriatric long term cares facilities 🞎 Day hospital 🞎 Dialysis 🞎 Intensive cares (adult or paediatric) 🞎 Other 🞎

Specify:

1. **Sample : collect of swab:**

Collected by: patient 🞎 nurse 🞎

Rectum 🞎 Stools 🞎 Ostomy 🞎

Diarrheal patient (≥ 3 stools /D during 48h)? Yes 🞎 No 🞎

1. **Have you been hospitalized abroad in the last 12 months:**

Yes 🞎 No 🞎 Don’t know 🞎

If yes: in which country: in which town:

Medical ward:

Medicine (adult or paediatric) 🞎 Surgery (adult or paediatric) 🞎 Obstetric 🞎 Non geriatric rehabilitation 🞎 Non geriatric long term cares facilities 🞎 Geriatric long term cares facilities 🞎 Day hospital 🞎 Dialysis 🞎 Intensive cares (adult or paediatric) 🞎 Other 🞎

Specify:

Duration of stay: less than a week 🞎 If more than one week: Number of weeks =

Date of end of stay:

1. **Have you been hospitalized in France in the last 12 months:**

Yes 🞎 No 🞎 Don’t know 🞎

If yes: county: Hospital name:

Hospitalization: > 24h 🞎 Day hospital 🞎 Ambulatory surgery 🞎

Medicine (adult or paediatric) 🞎 Surgery (adult or paediatric) 🞎 Obstetric 🞎 Non geriatric rehabilitation 🞎 Non geriatric long term cares facilities 🞎 Geriatric long term cares facilities 🞎 Day hospital 🞎 Dialysis 🞎 Intensive cares (adult or paediatric) 🞎 Other 🞎

Specify:

Duration of stay: less than a week 🞎 If more than one week: Number of weeks =

Date of end of stay:

**Have you travelled to a country outside mainland France in the last 3 months?**

Yes 🞎 No 🞎 Don’t know 🞎

If yes, country or French overseas departments n° 1: n°2: n°3:

Duration of stay (number of weeks):

Lodging:

All-inclusive: Yes 🞎 No 🞎 Don’t know 🞎

Hotel: Yes 🞎 No 🞎 Don’t know 🞎

Backpacker: Yes 🞎 No 🞎 Don’t know 🞎

Family/Friends: Yes 🞎 No 🞎 Don’t know 🞎

Feed: local market 🞎 Family 🞎 Hotel/restaurant 🞎

Diarrhea during the stay (≥ 3 stools /D during 48h)? Yes 🞎 No 🞎 Don’t know 🞎

Infection with antimicrobial treatment: Yes 🞎 No 🞎 Don’t know 🞎

1. **Member of the intra-family unit having had a contact outside metropolitan France in the last 3 months:**

- Has anyone who lives with you stayed abroad or in the DOM-TOMs in the previous 3 months?
  Yes 🞎 No 🞎 Don’t know 🞎

If yes, country/ French overseas departments:

- Have you hosted someone who usually lives abroad or in the DOM-TOMs in the previous 3 months?
  Yes 🞎 No 🞎 Don’t know 🞎
- If yes, country/ French overseas departments:

1. **Member of the intra-family unit hospitalized outside metropolitan France in the last 12 months:**

- Has anyone living under your roof been hospitalized abroad or in the French overseas departments in the previous 12 months?

Yes 🞎 No 🞎 Don’t know 🞎

If yes, country/ French overseas departments:

- Have you housed someone who has been hospitalized abroad or in the French overseas departments and territories in the previous 12 months?

Yes 🞎 No 🞎 Don’t know 🞎

If yes, country/ French overseas departments:

1. **Eating habits :**

Main cooking style:

European 🞎 Asian 🞎 North African🞎 Sub-Saharan 🞎

South American 🞎 Oriental 🞎 Fast food 🞎

Diet: Vegan 🞎 Vegetarian 🞎 Mixed 🞎

- Poultry consumption :

≥ once/day 🞎 ≥ once/week 🞎 ≥ once/month 🞎 never 🞎

- Beef consumption :

≥ once/day 🞎 ≥ once/week 🞎 ≥ once/month 🞎 never 🞎

- Lamb consumption :

≥ once/day 🞎 ≥ once/week 🞎 ≥ once/month 🞎 never 🞎

- Pork consumption :

≥ once/day 🞎 ≥ once/week 🞎 ≥ once/month 🞎 never 🞎

- Fish consumption :

≥ once/day 🞎 ≥ once/week 🞎 ≥ once/month 🞎 never 🞎

- Consumption of raw milk products:

≥ once/day 🞎 ≥ once/week 🞎 ≥ once/month 🞎 never 🞎

Main cooking style: Home-made 🞎 Industrial ready meals 🞎

- Corporate catering :

≥ once/day 🞎 ≥ once/week 🞎 ≥ once/month 🞎 never 🞎

- Restaurant :

≥ once/day 🞎 ≥ once/week 🞎 ≥ once/month 🞎 never 🞎

- Sytle of restaurant :

Various 🞎 American fast food 🞎 Italian🞎 Maghrebian 🞎

Middle Eastern (Lebanese, Turkish…)🞎 Asian 🞎 other 🞎

Consumption outside the retail circuit: Yes 🞎 No 🞎

If Yes: family production 🞎 Local producers 🞎 exotic grocery store 🞎

Child feeding method: Breastmilk 🞎 formula milk 🞎 industrial milk 🞎

Solid food 🞎 Parenteral nutrition 🞎 Gastric tube 🞎

1. **History of antibiotic therapy in the last 6 months including current hospitalization:**

Yes 🞎 No 🞎 Don’t know 🞎 If yes for which infection:

| Aminoglycoside |  | Glycopeptide |  | Quinolone |  |
| --- | --- | --- | --- | --- | --- |
| Cephalosporin |  | Penicilline |  | Sulfamide |  |
| Carbapenem |  | Penicilline + inhibitor |  | Metronidazole |  |
| Cycline |  | Macrolides |  |  |  |

**Other:**

**Not documented** 🞎

1. **Associated Comorbidity:**

Yes 🞎 No 🞎 Don’t know 🞎 If yes for which?

| Progressive cancer |  | Chronic respiratory failure |  |
| --- | --- | --- | --- |
| Progressive blood disease |  | organ transplant (__________________) |  |
| heart disease |  | marrow transplant |  |
| Chronic renal failure (Cl MDRD < 50) |  | HIV |  |
| Cirrhosis |  | Diabetes |  |
| Chronic dialysis |  | Other |  |
| Physical disability |  |  |  |

1. **Current medication intake:**

Antacid (Oméprazole, Inexium, Pariet…): Yes 🞎 No 🞎

Gastric dressing (Maalox, Gaviscon, Renie...): Yes 🞎 No 🞎

Laxatives: Yes 🞎 No 🞎

1. **Medication intake in the last 6 months:**

Antacid (Oméprazole, Inexium, Pariet…): Yes 🞎 No 🞎

Gastric dressing (Maalox, Gaviscon, Renie...): Yes 🞎 No 🞎

Laxatives: Yes 🞎 No 🞎

1. **History of MDRO and/or *C. difficile* colonization/infection:**

- Yes 🞎 No 🞎 Don’t know 🞎
- Known ESBL carrier patient 🞎 date of last positive sample:

ESBL species:

*E. coli* 🞎 *K. pneum* 🞎 *K. oxytoca* 🞎 *E. cloacae* 🞎 Other:

- Known carbapenemase-producing Enterobacterales (CPE) carrrier patient 🞎 date of last positive sample:

CPE species :

*E. coli* 🞎 *K. pneum* 🞎 *K. oxytoca* 🞎 *E. cloacae* 🞎 Other :

- Known *A. baumannii* Imipenem resistant carrier patient 🞎 date of last positive sample:
- Known MRSA carrier patient 🞎 date of last positive sample:
- Known VRE carrier patient 🞎 date of last positive sample:

*Clostridium difficile* infection (**asymptomatic carriage excluded**) 🞎 date of last positive sample:
